# Long-term complete radiological response to immunotherapy in a patient with metastatic NSCLC and active rheumatoid arthritis

**DOI:** 10.1093/omcr/omag100

**Published:** 2026-06-15

**Authors:** Eleanor Roy, Amir Arshia Emam Jomeh, Eleni Xenophontos, Haris Charalambous

**Affiliations:** The University of Nicosia Medical School, Nicosia 2417, Cyprus; The University of Nicosia Medical School, Nicosia 2417, Cyprus; The University of Nicosia Medical School, Nicosia 2417, Cyprus; Bank of Cyprus Oncology Center, Nicosia 1680, Cyprus; Bank of Cyprus Oncology Center, Nicosia 1680, Cyprus

**Keywords:** NSCLC, rheumatoid arthritis, immunotherapy, autoimmune disease, checkpoint inhibitors

## Abstract

The use of immune checkpoint inhibitors (ICIs) in patients with autoimmune diseases (AIDs) is challenging due to the risk of immune-related adverse events and disease flares. These patients are routinely excluded from clinical trials, leaving limited evidence to guide practice. We report a 70-year-old woman with metastatic non-small cell lung cancer (NSCLC) and active rheumatoid arthritis (RA) receiving infliximab and methotrexate. Initial treatment with platinum-based chemotherapy excluded ICIs. After disease progression, pembrolizumab was introduced with chemotherapy following multidisciplinary discussion. The patient achieved a complete radiological response without RA flare, completed two years of pembrolizumab in March 2022, and remains in remission as of August 2025. This case highlights that ICIs can be used safely in selected patients with active AID, preserving functional status and quality of life. Prospective studies in this population are needed to refine risk–benefit assessment and optimise patient selection.

## Introduction

Immune checkpoint inhibitors (ICIs) targeting cytotoxic T-lymphocyte-associated protein 4 (CTLA-4), programmed death-1 (PD-1), and programmed death-ligand 1 (PD-L1) have transformed the management of non-small cell lung cancer (NSCLC) by enhancing T-cell–mediated antitumour responses [1]. Agents, such as pembrolizumab, nivolumab, and atezolizumab, have demonstrated significant survival benefits, particularly in patients with metastatic NSCLC lacking actionable molecular aberrations, and are effective irrespective of PD-L1 expression levels [2].

However, the use of ICIs in patients with pre-existing autoimmune diseases (AIDs) presents clinical challenges. AIDs involve the breakdown of self-tolerance and immune dysregulation, increasing the risk of immune-related adverse events (irAEs) and disease flares [1]. Consequently, these patients are excluded from most trials, leaving little high-quality data to guide management [3]. Retrospective studies have reported flares in up to 38% and de novo irAEs in 20%–30% of patients with AIDs receiving ICIs, most of which are manageable but occasionally severe [3]. Given that up to 25% of patients with NSCLC have underlying AIDs, this represents a significant evidence gap [4]. Recent systematic reviews and meta-analyses suggest that patients with pre-existing autoimmune disease experience higher rates of autoimmune flares and de novo immune-related adverse events; however, most events are low grade, and severe (grade ≥ 3) toxicity is uncommon and often manageable with corticosteroids and/or temporary interruption of immunotherapy [5, 6].

Rheumatoid arthritis (RA), among the most common AIDs in oncology, is typically managed with corticosteroids, non-steroidal anti-inflammatory drugs (NSAIDs), and disease-modifying anti-rheumatic drugs (DMARDs), such as methotrexate and antitumour necrosis factor (anti-TNF) agents.

This case report describes a patient with metastatic NSCLC and active RA receiving DMARD therapy who achieved a durable clinical response to pembrolizumab without autoimmune exacerbation, offering real-world insight into the feasibility of ICI treatment in this complex subgroup.

## Case report

A 70-year-old woman with seropositive rheumatoid arthritis (RA, diagnosed 2013) presented in August 2019 with haemoptysis. Computed tomography (CT) revealed a left lower lobe mass, and bronchoscopy confirmed lobar occlusion. Biopsy showed lung adenocarcinoma (TTF-1 and Napsin A positive; p63 negative). She reported non-productive cough, exertional dyspnoea, malaise, and diffuse arthralgia, without weight loss or appetite changes.

**Figure 1:**
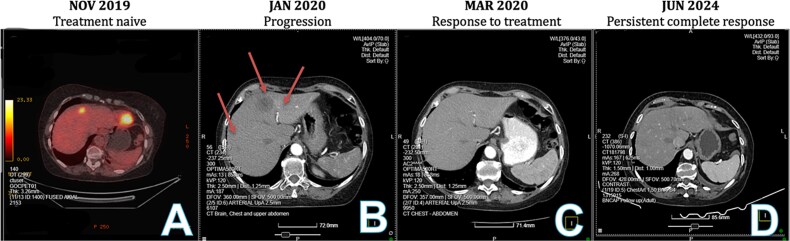
Image showing liver metastases evolution: (A) baseline imaging at the time of diagnosis from FDG-PET/CT showing two liver lesions; (B) computed tomography (CT) showing disease progression with the presence of new lesions (red arrows) from contrast-enhanced CT abdomen; (C) CT abdomen showing complete response (CR); (D) CT abdomen showing persistent CR.

Staging brain CT was unremarkable, while abdominal imaging revealed two hepatic metastases. FDG-PET/CT confirmed mediastinal invasion, lymphadenopathy, lobar obstruction, suspected lymphangitic carcinomatosis, two liver lesions (25 mm and 20 mm), and a thoracic vertebral metastasis, consistent with cT4N2M1c (8th edition) (Fig. 1A).

At the time of diagnosis in November 2019, she was receiving infliximab (anti-TNF), methotrexate (7.5 mg weekly), and folic acid for RA. Infliximab was discontinued given its association with cancer progression [7], while methotrexate and folic acid were continued in consultation with her rheumatologist. Molecular profiling (ONCOMINE FOCUS) revealed no actionable mutations; PD-L1 expression was 75% (22C3 clone). Her Eastern Cooperative Oncology Group performance status (ECOG PS) at diagnosis was 1.

Because of active RA and recent anti-TNF exposure, first-line treatment was platinum–pemetrexed chemotherapy without immunotherapy, deferring pembrolizumab in line with the KEYNOTE-189 regimen [8]. After three cycles, imaging in January 2020 showed disease progression (Fig. 1B). Pembrolizumab was then introduced alongside carboplatin–paclitaxel following multidisciplinary discussion with oncology and rheumatology. As the patient had not received infliximab for ~ 3 months and she did not have any extra-articular disease since RA diagnosis, pembrolizumab was considered a safe option. Assessment at each cycle for new joint pain, swelling, stiffness, or functional decline, with periodic ESR/CRP and early rheumatology review (within ~ 4–8 weeks of initiation) was introduced as a mitigation plan. This combination produced a complete radiological response in pulmonary and hepatic lesions without RA flare (Fig. 1C). After six cycles, chemotherapy was stopped and pembrolizumab continued as maintenance. During this period, there was no change in her RA treatment, as there were no flares.

During maintenance, she developed mild (grade 1–2) arthralgia, mainly affecting her hands, without extra-articular manifestations; this was managed with prednisolone 5 mg daily. She preserved an ECOG PS 1 throughout treatment. She completed two years of pembrolizumab in March 2022 and remains in remission in August 2025, with stable RA and no relapse (Fig. 1D). A timeline summarizing oncologic therapy, RA treatment, and outcomes is provided in Table 1.

**Table 1:** Timeline of oncologic treatment, rheumatoid arthritis therapy, imaging response, and immune-related events.

Date	Cancer Treatment	RA Treatment	Imaging/Clinical Outcome	Flares/irAEs
Aug 2019	-	Infliximab + methotrexate + folic acid (pre-existing RA)	Presentation with haemoptysis; work-up initiated	-
Nov 2019	Start platinum–pemetrexed chemotherapy (ICI deferred)	Infliximab discontinued; methotrexate + folic acid continued	Molecular profiling: no actionable mutations; PD-L1 75%; baseline metastatic disease (lung + liver + bone)	Not applicable (pre-ICI baseline)
Jan 2020	After 3 cycles: disease progression → switch to carboplatin–paclitaxel + pembrolizumab (post-MDT oncology/rheumatology)	Methotrexate + folic acid continued; mitigation plan: symptom checks each cycle + periodic ESR/CRP + early rheumatology review	Imaging confirmed progression pre-switch; then treatment initiated	No RA flare; no new clinically significant irAEs reported
2020 (after 6 cycles)	Chemotherapy stopped; pembrolizumab continued as maintenance	No change in RA regimen (no flares)	Complete radiological response in pulmonary and hepatic lesions	Mild arthralgia (grade 1–2)
2020–2022	Pembrolizumab maintenance continued (total planned 2 years)	Prednisolone 5 mg daily for arthralgia; RA otherwise stable	Sustained complete response; ECOG PS remained 1 throughout treatment	No RA flare; mild arthralgia only
Mar 2022	Pembrolizumab completed (2 years)	RA stable on ongoing therapy	Ongoing remission at completion	No grade ≥ 3 irAEs; mild arthralgia only
Aug 2025	-	RA stable	Remains in remission (complete response maintained)	No flare/late irAEs reported

## Discussion

Patients with pre-existing autoimmune disease (AID), including rheumatoid arthritis (RA), were largely excluded from pivotal ICI trials, so evidence comes mainly from retrospective cohorts and meta-analyses [5, 9]. The main concern is AID flare and development of new irAEs. However, systematic reviews and meta-analyses comparing patients with AID with patients without AID show that patients with AID have higher risk of any irAEs leading to ICI discontinuation, but no clear increase in severe irAEs, PFS and OS [5, 9].

This case illustrates the feasibility of immune checkpoint inhibitor (ICI) therapy in a patient with metastatic NSCLC and active RA on disease-modifying therapy, who achieved a durable complete response without significant autoimmune toxicity.

Evidence from a recent systematic review of 95 retrospective studies (n = 23 897) found that patients with pre-existing autoimmune disease (AID) treated with ICIs had a higher risk of immune-related adverse events (irAEs) (RR 1.3). Overall, 61% experienced irAEs, including 36% AID flares and 23% de novo irAEs; most were grade 1–2. About 32% required hospitalisation, 72% received corticosteroids, and irAE-related mortality was low (0.07%). Lung cancer accounted for 30.7% of the cohort [5].

A meta-analysis of 24 retrospective cohorts involving 11 567 patients (3774 with NSCLC; 1157 with AID) found that NSCLC patients with AID had higher rates of de novo irAEs (40%) but fewer flares (23%) than the broader cancer population. Importantly, NSCLC patients who developed irAEs also had an increased risk of severe irAEs (grade 3–4) and an improved radiological response (RR 1.56), supporting the hypothesis that irAEs reflect stronger antitumour immunity [4].

In rheumatological AIDs specifically, a meta-analysis of 643 patients reported flares in 41% and de novo irAEs in 33%**.** Severe events were observed in 7% of flares and 12% of irAEs. Patients with RA had a greater risk of flare (RR 1.35), though baseline DMARD use was not associated with higher flare rates or reduced response [6].

The risks of ICI use vary by AID type, severity, and immunosuppressive regimen. Rheumatologic AIDs may be safer than neurological ones such as myasthenia gravis or Guillain–Barré, where flares are life-threatening and ICIs avoided. Real-world series are likely biased toward patients with less severe AID, as those on anti-TNF agents are usually considered at higher risk and excluded from treatment [3].

Not all patients with autoimmune disease, including rheumatoid arthritis, develop flares during immune checkpoint inhibitor therapy because autoimmune conditions are heterogeneous in activity and immune pathways, and many patients begin treatment in remission or on background DMARD therapy that helps maintain immune tolerance. In addition, checkpoint blockade mainly enhances tumour-specific T-cell responses while other regulatory mechanisms remain intact, and individual genetic, microbiome, and treatment-related factors (such as type of ICI or combination therapy) further influence whether a clinically significant autoimmune flare occurs.

Tumour features also guide selection. Patients with high PD-L1 expression (>50%) derive greater benefit, as demonstrated in KEYNOTE-042 [10]. At the time of her diagnosis, pembrolizumab was the only immune checkpoint inhibitor approved in the metastatic lung cancer setting in Cyprus. In contrast, oncogene-driven NSCLC (EGFR, ALK, HER2) rarely benefits from ICIs, as shown in the IMMUNOTARGET registry [11].

Overall, these data support individualised, multidisciplinary risk–benefit assessment when considering ICIs in patients with RA. Prospective studies that include patients with autoimmune diseases, stratified by disease type, severity, DMARD use, and biomarkers such as PD-L1, and next-generation sequencing results, are needed to provide more definitive guidance [5, 6, 9].

## Conclusion

This case adds to the real-world evidence supporting the safe use of ICIs in NSCLC patients with autoimmune comorbidities such as RA. Despite their exclusion from pivotal trials, our patient achieved a durable complete response with pembrolizumab and remains in remission more than three years after treatment.

With multidisciplinary planning and close monitoring, immunotherapy can be delivered safely even in patients on disease-modifying treatment. Prospective studies including patients with autoimmune diseases, stratified by type, severity, and biomarkers, are needed to guide practice.

Patients who remain relatively stable with their autoimmune disease without systemic and frequent flares could be such candidates. For similar patients, a pragmatic approach is oncology–rheumatology MDT co-management, documentation of baseline autoimmune activity and functional status, shared decision-making, and an agreed monitoring and flare-management pathway prior to initiating ICIs.

## Declarations

Dr H. Charalambous declares institutional research funding and travel expenses from MSD. The authors declare no conflicts of interest.

Written informed consent was obtained from the patient for publication of this case report.

Ethical approval was not required for this case report.
